# ABCD transfer matrix model of Gaussian beam propagation in plano-concave optical microresonators

**DOI:** 10.1364/OE.484212

**Published:** 2023-05-03

**Authors:** David Martin-Sanchez, Jing Li, Edward Z. Zhang, Paul C. Beard, James A. Guggenheim

**Affiliations:** 1 Department of Medical Physics and Biomedical Engineering, University College London, UK; 2 Wellcome / EPSRC Centre for Interventional and Surgical Sciences, University College London, UK; 3 Institute of Cardiovascular Sciences, College of Medical and Dental Sciences, University of Birmingham, UK; 4 School of Engineering, College of Engineering and Physical Sciences, University of Birmingham, UK

## Abstract

Plano-concave optical microresonators (PCMRs) are optical microcavities formed of one planar and one concave mirror separated by a spacer. PCMRs illuminated by Gaussian laser beams are used as sensors and filters in fields including quantum electrodynamics, temperature sensing, and photoacoustic imaging. To predict characteristics such as the sensitivity of PCMRs, a model of Gaussian beam propagation through PCMRs based on the ABCD matrix method was developed. To validate the model, interferometer transfer functions (ITFs) calculated for a range of PCMRs and beams were compared to experimental measurements. A good agreement was observed, suggesting the model is valid. It could therefore constitute a useful tool for designing and evaluating PCMR systems in various fields. The computer code implementing the model has been made available online.

## 1. Introduction

Plano-concave microresonators (PCMRs) are optical devices consisting of one planar and one concave mirror separated by a spacer to form a planoconcave optical cavity [1]. In principle, this geometry allows for a near perfect confinement of a focused laser beam, providing a cavity with a high Q factor. This is of interest in a range of applications, including quantum electrodynamics metrology [2–4], temperature and gas pressure sensing [5–7], and ultrasonic detection in photoacoustic imaging [8,9].

A key characteristic of a PCMR is the wavelength-resolved interferometer transfer function (ITF). The ITF quantifies the light intensity reflected by the PCMR as a function of wavelength. Due to the resonant nature of the PCMR, the ITF exhibits a series of regularly spaced spectral peaks referred to as fringes. The characteristics of these fringes determine the device performance. For example, in PCMR sensors used to measure ultrasound, the PCMR undergoes thickness changes in response to ultrasonic waves, leading to spectral shifts in the ITF. These shifts are measured by monitoring the changes in the light intensity reflected by the PCMR, at a wavelength at the edge of a fringe. Under these conditions, the gradient of the fringe determines the sensitivity of the sensor. In other PCMR-based devices such as filters, characteristics such as the fringe width are of primary interest. Predicting ITFs by modelling the propagation of light in PCMRs could therefore aid the design and evaluation of a range of PCMR based devices.

Existing methods allow predicting certain optical characteristics of PCMRs. For example, simple calculations can predict the curvature required for the PCMR’s spherical mirror to best confine a given illumination beam [10,11]. Analytical methods can also determine the coupling efficiency of light into different cavity modes by solving the overlap integrals between the input and resonating fields [12,13]. Numerical methods, such as the Fox-Li method [14,15], also enable performing modal analysis and studying mode stability by solving the Maxwell’s equations. Other methods allow predicting the transmitted or reflected field in certain conditions, enabling calculating ITFs in those conditions. For example, the well-known Airy Function [16] can predict the ITF if the radius of curvature (ROC) of the spherical mirror of the PCMR matches the curvature of the wavefront of the beam [17]. In these conditions, the beam propagation is analogous to that of a plane wave in a planar Fabry-Perot (FP) etalon. Similarly, if the ROC of the PCMR is near infinite, the PCMR effectively is a planar etalon. In these conditions, the angular Airy Function (AAF) [18], a numerical model that decomposes any beam into plane waves, can be used to predict the ITF for any beam. While these methods are useful in certain cases, they do not enable calculating the ITF for PCMRs and beams of arbitrary geometry. To overcome this limitation, some AAF-based methods have represented spherical mirrors as non-uniform 2D arrays of phase shifts to account for the wavefront distortions encountered by the beam [19,20]. However, this approach is computationally expensive. Therefore, there is a need for a more broadly applicable method for efficiently predicting the ITFs of PCMRs.

To address this need, a model of Gaussian beam propagation through PCMRs was developed. The model is based on the ABCD method [21]. This is a well-established method for propagating paraxial beams through optical systems comprising passive components such as lenses and mirrors [22–24]. The propagation of light through each component is represented by an ABCD matrix. By multiplying the individual component matrices together, a single matrix representing the whole system can be formulated. It is also possible to model resonant optical devices by iteratively applying ABCD matrices. This approach was previously applied to model planar FP etalons (microcavities comprising two plane-parallel mirrors [25,26]), which exhibit poor beam confinement when illuminated by tightly focused Gaussian beams. Here, it is applied to model PCMRs, which can be designed to exploit better beam confinement and produce ITFs with different characteristics. In the new model, ABCD matrices representing a PCMR and the optical systems delivering the beam to and from the PCMR are defined and multiplied to form a system matrix. This matrix is used to iteratively compute the partial fields generated by the multiple roundtrips that light undergoes in the PCMR. The partial fields are summed to obtain the total optical field reflected by the PCMR. Finally, the ITF is calculated by spatially integrating the field and repeating the process for multiple wavelengths. To validate the model, the predicted ITFs of PCMRs were compared to those calculated using Airy functions where applicable, and experimentally measured ITFs.

The following sections describe the principles of the model and its implementation, the experimental methods used to measure ITFs, and the results in terms of the predicted and measured ITFs and their agreement. These sections are followed by discussions and conclusions.

## 2. Model

This section describes the ABCD model of light propagation in PCMRs. First, the ABCD method is introduced. Then, the methods for calculating the ABCD matrices, the total reflected fields, and the ITFs of a PCMR system are explained.

### 2.1 Using the ABCD method

The ABCD method can be used to simulate the propagation of paraxial beams such as plane waves and Gaussian beams through a system comprising multiple optical components. Each component is described by a 2-by-2 transfer matrix of the form *M = [A B; C D]* (a list of examples can be found in Ref. [1]). The matrix encodes the transformation to be applied to an incident beam, *U_in_*, propagating through the component along the optical axis, *z*. The transformation is given by the ABCD law [27]

(1) q(z1)→[ABCD]→q(z2)=Aq(z1)+BCq(z1)+D,

where *z_1_* and *z_2_* are the planes located immediately before and after the optical system, and *q* is the complex beam parameter encoding the beam parameters at a plane normal to the optical axis. For a Gaussian beam *q* is defined by

(2) $$\frac{1}{q(z)}=\frac{1}{R(z)}-i\frac{2}{k\omega^{2}(z)},$$

where *R(z)* is the ROC of the wavefront, *ω(z)* is the transverse *1/e^2^* beam width, *k = 2πn/λ* is the wave number, *n* is the refractive index of the medium, and *λ* is the wavelength of the beam. The complex amplitude of the field, *U_out_*, across the radial axis, *r*, at a position *z* along the optical axis, is given by [12]

(3) $$U(r,z)=\frac{\omega_{0}}{\omega (z)}\exp\left(-\frac{r^{2}}{\omega^{2}(z)}\right)\exp\left(-ikz-\frac{ikr^{2}}{2R(z)}-i\zeta (z)\right),$$

where *ς(z)* is the Gouy phase, *z_0_* is the Rayleigh range, and *ω_0_* is the beam width at the focus.

As ABCD matrices are linear operators, the system matrix representing an optical system comprising any number of optical components can be found by multiplying the matrices of the constituent components together. Eqs. (1-3) can then be used to calculate the profile of a beam propagated through the system.

### 2.2 Defining the PCMR matrix

**Fig. 1.**
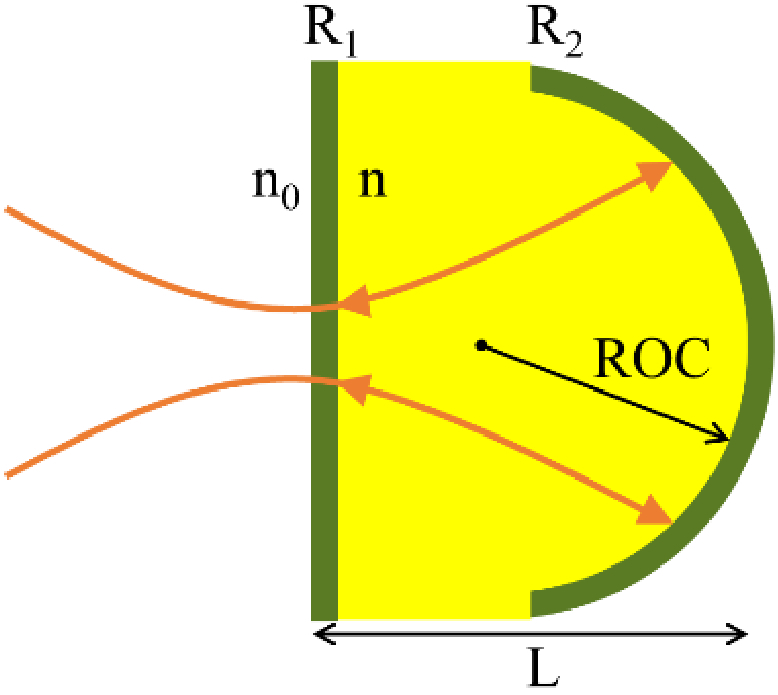
Schematic of a PCMR and the beam path after one reflection on the concave mirror.

A PCMR consists of two mirrors of reflectivity *R_1_* and *R_2_*, separated by a spacer of refractive index *n* and thickness *L* [Fig. 1]. The first mirror is flat and parallel to the plane of incidence, while the second mirror has a spherical shape of given ROC.

A Gaussian beam, *U_in_*, incident upon the first (planar) mirror of the PCMR is partly reflected and partly transmitted into the cavity. The transmitted light undergoes an integer number of round trips, *m*, inside the cavity, generating multiple partial reflected beams, *U_m_*, that exit the cavity. The total reflected beam is determined by the interference of these partial beams and the initial reflection from the first planar surface.

To represent a PCMR using an ABCD matrix, three sub-matrices are needed to propagate the beam: (1) into the cavity, *M_in_*; (2) through a round trip, *M_cav_*; and (3) out of the cavity, *M_out_*. These operations can each be represented using a well-known existing ABCD matrix [1] as follows. The transmission of the incident beam, from a surrounding medium of refractive index *n_0_*, into the cavity is represented by a matrix representing refraction at a planar boundary

(4) $$M_{in}=M_{\mathrm{refr}}=\left[\begin{array}{cc} 1 & 0\\ 0 & n_{0}/n \end{array}\right].$$

The roundtrip matrix is formed by multiplying matrices representing a propagation through space (the cavity), reflection by a spherical mirror, another propagation through space (back through the cavity), and a reflection at a planar mirror,

(5) $$M_{\text{cav}}={M_{\text{refl}}}^{\leftarrow }{M_{\text{prop}}}^{\leftarrow }M_{\text{sph}\_\text{refl}}M_{\text{prop}}=\left[\begin{array}{cc} 1 & 0\\ 0 & 1 \end{array}\right]\left[\begin{array}{cc} 1 & L/n\\ 0 & 1 \end{array}\right]\left[\begin{array}{cc} 1 & 0\\ -2/ROC & 1 \end{array}\right]\left[\begin{array}{cc} 1 & L/n\\ 0 & 1 \end{array}\right].$$

Note that where the beam is propagating opposite to the incident beam, *M^←^*, the Reverse Propagation Theorem [28] has been applied.

The exit matrix is defined in the same way as the roundtrip matrix, except that the matrix applied last is replaced with one representing refraction, rather than reflection, by the planar mirror,

(6) $$M_{\text{out}}={M_{\text{refr}}}^{\leftarrow }{M_{\text{prop}}}^{\leftarrow }M_{\text{sph}\_\text{refl}}M_{\text{prop}}.$$

Combining these three matrices together, yields the ABCD matrix representing the PCMR

(7) $$M_{PCMR,m}=M_{\text{out}}{\left(M_{\text{cav}}\right)}^{m}M_{\text{in}}.$$

Equation (7) can be generalised to represent the propagation of the incident beam through an optical system containing a PCMR and any optical components (e.g., lenses) used to deliver the beam to and from it,

(8) $$M_{\text{syst},m}=M_{\text{detection}}M_{PCMR,m}M_{\text{illumination}},$$

where *M_illumination_* represents the optical components between the illumination source and the PCMR and *M_detection_* represents the components between the PCMR and the detector. Equation (8) now represents the propagation of a single partial beam from a source to the detector, via a PCMR, having undergone exactly *m* round trips inside it.

### 2.3 Calculating the total reflected field

Once a PCMR system is described by an ABCD matrix as defined above [Eq. (8)], the complex amplitude of each partial beam, *U_m_*, can be calculated using Eqs. (1-3). The total reflected field, *U_out_*, can then be obtained by summing the partial beams and the initial reflection, while accounting for the transmission and reflection coefficients of the mirrors and the absorption coefficient of the spacer, *µ_a_*,

(9) $$U_{\text{out}}=r_{1}U_{in}+\sum_{m=0}^{\infty }A_{m}U_{m}\sqrt{\exp(-\mu_{a}2(m+1)L)}\;A_{m}=t_{1}r_{2}t_{1}{\left(r_{1}r_{2}\right)}^{m},\;m\geq 0$$

where *r_1_* and *r_2_* are the reflection coefficients of the first and second mirror, respectively, and *t_1_* is the transmission coefficient of the first mirror.

### 2.4 Calculating the ITF

The ITF is defined as

(10) $$ITF(\lambda )=\frac{I_{\text{out}}(\lambda )}{I_{\text{in}}(\lambda )},$$

where *I_in_* and *I_out_* are the optical intensities of the field measured before and after the light has propagated through the optical system to the detector. Computing *I_out_* requires appropriately spatially integrating the field to account for the detection method. For example, if the detector is uniformly sensitive and larger than the beam (thus effectively infinite in extent) and sensitive only to intensity, then [29]

(11) $$I_{\text{out}}=\overset{\infty }{\underset{0}{\int }}{\left|U_{\text{out}}(r,z)\right|}^{2}2\pi rdr.$$

Equally, if the light is delivered to the detector via a single mode fibre, then [30]

(12) $$I_{\text{out}}={\left|\overset{\infty }{\underset{0}{\int }}U_{\text{out}}(r,z)\exp\left(-\frac{4r^{2}}{D^{2}}\right)2\pi rdr\right|}^{2},$$

where *D* is the mode field diameter of the fibre.

## 3. Experimental methods

This section describes the methods used to measure the ITFs of PCMRs in order to validate the presented model.

### 3.1 Experimental system

**Fig. 2.**
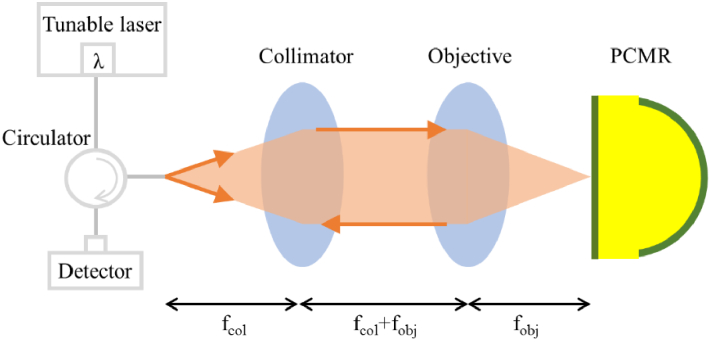
Schematic diagram of the experimental optical system.

The system used to measure ITFs is shown in Fig. 2. The system was similar to that described in Ref. [31]. Briefly, a PCMR was illuminated by a laser beam provided by a tunable continuous-wave laser (Tunics T100S-HP/SCL, Yenista Optics) with a wavelength range of 1440-1640 nm. To deliver the beam, the laser light was coupled into a single mode fibre with a mode field diameter of 10.4 ± 0.5 µm. After exiting the fibre, the beam was focused onto the PCMR by a series of lenses selected to produce a beam with a given focal beam waist located at the planar mirror of the PCMR. The reflected beam was coupled back into the fibre, and delivered by an optical circulator (6015-3, Thorlabs) to a custom-designed AC and DC-coupled InGaAs photodiode (G9801-22, Hamamatsu) and amplifier unit [31]. The light intensity at the detector was recorded to a computer using a data acquisition card (PCI-6229, National Instruments).

### 3.2 PCMR

**Fig. 3.**
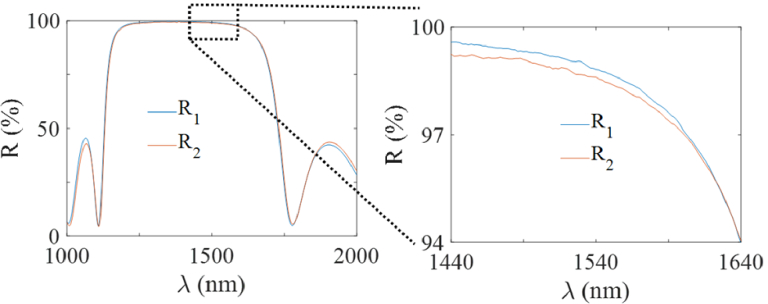
Measured reflectivity of the flat (R_1_) and spherical (R_2_) mirror coatings. The graph on the right is a zoomed-in version of the reflectivities over the operating wavelength range of the tunable interrogation laser used.

The PCMR under test was custom designed with two dielectric mirrors (one planar, one concave), a fused silica spacer of thickness L = 1.2 mm, a ROC of 2.5 mm, and a diameter of 140 µm. To enable measuring ITFs at different reflectivities, the mirrors were designed to have spectrally varying reflectivities. Changing the interrogation laser wavelength thus allowed the PCMR mirror reflectivities to be varied. The mirror reflectivities are plotted in Fig. 3. The reflectivity is in the range 94-99.2% in the wavelength range 1440-1640 nm.

### 3.3 PCMR alignment

The system was aligned so that the beam waist was normally incident on the first mirror and centred on the PCMR axis. To achieve this, a series of translation and tip-tilt stages were used, first to adjust the axial position and angle of the PCMR to maximise the reflected optical power, then to centre the PCMR on the beam. To ensure the beam was correctly aligned over the wavelength range of interest, the optical wavelength used for alignment, *λ_a_*, was chosen to be close to the wavelengths of interest, but away from any fringe to ensure the PCMR acted only as a planar mirror during the alignment procedure.

### 3.4 ITF measurement and analysis

To acquire the ITF, the PCMR was aligned as described above. The laser wavelength was then scanned over the range of interest, and the light intensity on the photodiode recorded as a function of the wavelength. To determine the intrinsic ITF of the PCMR, the measured intensity was normalised by an empirically determined spectrally varying scaling factor representing variations in the optical power delivered through the system [32].

To analyse the ITF, four metrics were calculated for each measured fringe. These were: (1) the free spectral range (FSR), denoting the fringe spacing; (2) the full width at half maximum (FWHM), quantifying the fringe width; (3) the finesse, *F = FSR/FWHM*; and (4) the visibility, *V=|I_max_-I_min_|/(I_max _+ I_min_),* where *I_min_* and *I_max_* are the minimum and maximum optical intensity of the resonant fringe.

## 4. Results

The ABCD PCMR model was validated in two steps. In the first step, predicted ITFs were compared to those predicted by an AAF model of planar FP etalons [18]. In the second step, ITFs predicted by the ABCD model were compared to experimentally measured PCMR ITFs. Note that in all cases where ITFs were predicted by the ABCD model, the computation of the infinite number of partial beams to obtain the total reflected beam was approximated by a finite sum, as explained elsewhere [25]. Briefly, this approximation uses an appropriate number of partial beams (roundtrips) which is determined using a convergence criterion, based on the change in the total field profile due to the latest round trip (once the sum of the change was <0.001%, no further round trips were calculated).

### 4.1 Comparisons with an AAF based model

ITFs predicted by the ABCD model were compared to those predicted by an AAF model [18] in two scenarios in which the ITF of the PCMR was expected to be identical to that of a planar etalon. In the first scenario, the ROC of the spherical mirror in the PCMR and the ROC of the beam wavefront were equal, i.e.

(13) $$ROC_{mirror}=ROC_{beam}=L\left(1+{\left(\frac{k\omega_{0}^{2}}{2L}\right)}^{2}\right).$$

In this scenario, the beam confinement is expected to be analogous to that of a collimated beam in a planar etalon. As such, the ITF of the PCMR is expected to equal that of an FP etalon illuminated by a large diameter beam. In the second scenario, the ROC of the PCMR was infinite, such that the PCMR was identical to a planar FP etalon.

**Fig. 4.**
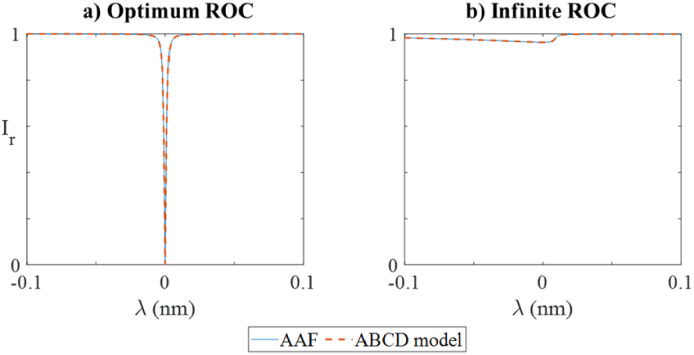
Simulated ITFs of a PCMR (*L *= 1200 µm, *n *= 1.444, *R_1 _*= *R_2 _*= 98%) using the ABCD model (dashed lines) where: a) ROC of second mirror matches curvature of illuminating Gaussian beam with spot size 2*ω_0 _*= 38 µm and *λ* near 1550 nm, compared to AAF simulation (solid line) of same resonator but two planar mirrors illuminated by collimated beam (2*ω_0 _*= 3800 µm); and b) ROC is infinite and same beam as in (a), compared to AAF simulation (solid line) of same FP etalon illuminated by same beam.

To perform the investigation, an ABCD model was constructed as described in section 2 for a PCMR illuminated by a beam with a focal waist of 38 µm. The characteristics of the PCMR were as described in section 3, except that the ROC was equal to that of the interrogation beam [as in Eq. (13)]. The ITF was calculated using Eq. (10) and Eq. (12) and plotted in Fig. 4(a). For comparison, the ITF of the same planar etalon illuminated by a beam with a large focal waist of 2ω_0 _= 3800 µm (approximating a plane wave) was calculated using the AAF and plotted in Fig. 4(a). A good agreement was observed between the two ITFs.

The investigation was repeated with the ABCD matrices representing the PCMR adapted to provide a spherical mirror of infinite ROC, effectively yielding a planar FP etalon. Resulting ITFs predicted by the ABCD and AAF models are plotted in Fig. 4(b). The ITFs are in excellent agreement. The low finesse and low visibility are due to the significant curvature mismatch between the beam and the spherical mirror, which provides a poor beam confinement.

### 4.2 Comparisons with experimental measurements

To experimentally validate the PCMR model, predicted and measured ITFs were compared in three sets of experiments involving (1) well-matched ROCs, (2) poorly matched ROCs and (3) defocused interrogation beams.

#### 4.2.1 Well matched beam

**Fig. 5.**
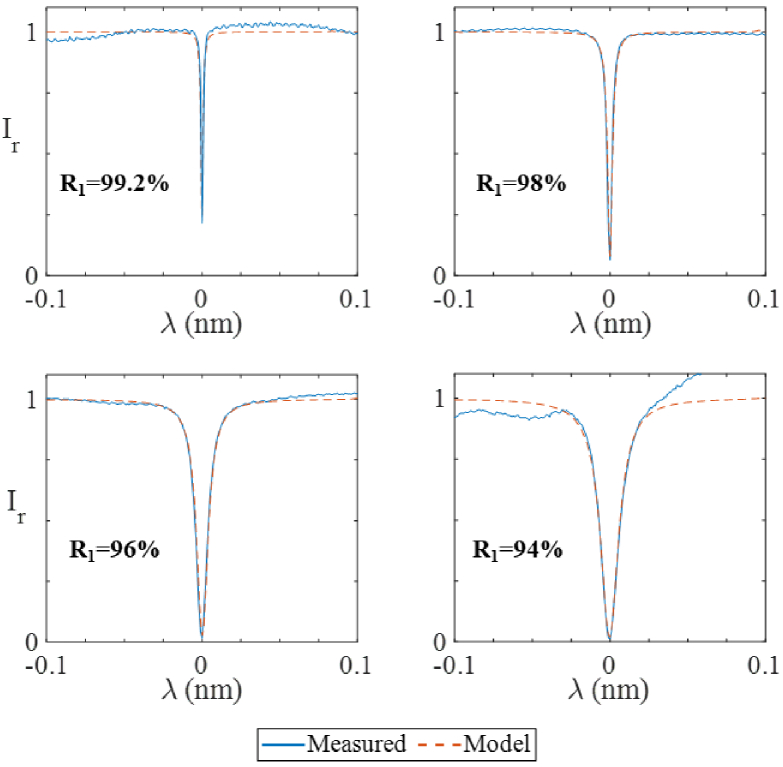
Comparison of ITFs simulated with the ABCD model (dashed lines) and experimentally measured (solid lines) for different first mirror reflectivities (*R_1_*) and a focused interrogation beam of 2*ω_0 _*= 38 µm.

In the first set of experiments, the PCMR, whose ROC (2.5 mm) perfectly matched a beam of 2*ω_0 _*= 41 µm, was interrogated by a beam of 2*ω_0 _*= 38 µm, which is considered a good match. More than 300 fringes were acquired over the 1440-1640 nm wavelength range of the interrogation laser, each of which represents the ITF of a PCMR with a different mirror reflectivity. A representative subset of the ITFs for *R_1 _*= 94%, 96%, 98% and 99.2% reflectivity, are plotted in Fig. 5, along with the corresponding measured ITFs for comparison. In all cases, but for minor variations in the baseline values, there is excellent agreement between the measured and predicted fringes.

**Fig. 6.**
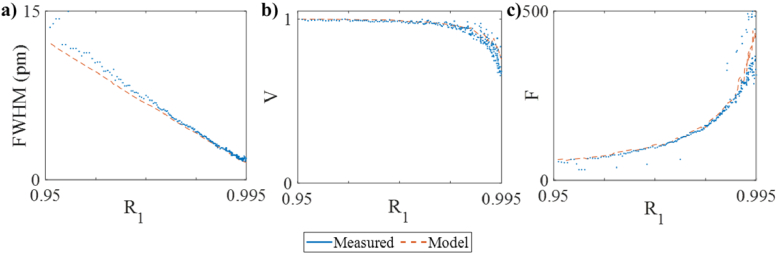
Comparison of (a) full width at half maximum (FWHM); (b) visibility, V=|Imax-I_min_|/(I_max _+ I_min_); and (c) finesse, F = FSR/FWHM, where FSR is the free spectral range, experimentally measured (dots) and simulated with the ABCD model (dashed lines) using 2*ω_0 _*= 38 µm.

To provide a comprehensive quantitative comparison, the ITFs were evaluated in terms of their FWHM, visibility and finesse. These metrics were then plotted as a function of the first mirror reflectivity (*R_1_*) in Fig. 6. As shown in Fig. 6(a) the FWHM of the predicted ITFs decreases with increasing reflectivity, from about 15 nm at *R_1 _*= 95% to about 1.5 pm at *R_1 _*= 99.5%. The measured FWHMs are in close agreement, with a root mean square error below 0.5 pm. The visibility of the predicted ITFs is approximately 1 for *R_1 _*< 98%. [Figure 6(b)]. It then decreases increasingly rapidly as the reflectivity increases, down to a minimum value of about 0.6 at *R_1 _*= 99.5%. The measured visibilities are in close agreement with these values, with a root mean square error below 0.03. The finesse [Fig. 6(c)] of the predicted ITFs increases with increasing reflectivity, from about 30 at *R_1 _*= 95% to about 500 at *R_1 _*= 99.5% and again is in agreement with the experimentally measured finesse with a root mean square error below 20.

#### 4.2.2 Poorly matched beam

**Fig. 7.**
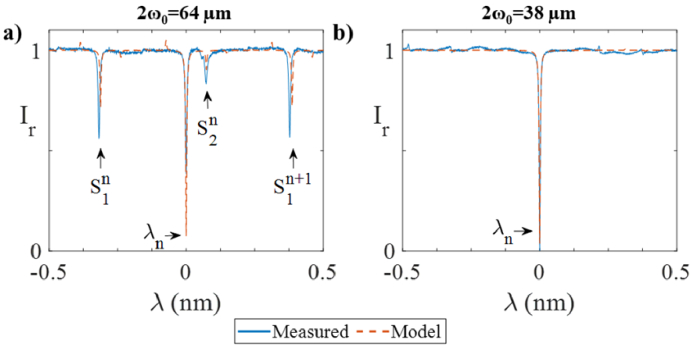
Effect of mismatch between interrogation Gaussian beam and PCMR ROC. Reflected ITF simulated with the ABCD model (dashed lines) and experimentally measured (solid lines) using (a) 2*ω_0 _*= 64 µm and (b) 2*ω_0 _*= 38 µm (*λ_a_* = 1580 nm). The n^th^ main fringe is denoted as *λ_n_* and the n^th^ set of secondary fringes as *S_i_^n^*, where *i *= 1,2.

The measurements were repeated using a different beam of 2*ω_0 _*= 64 µm, which was poorly matched to the 2.5 mm ROC of the PCMR. A representative example of a resulting ITF is plotted in Fig. 7(a), along with the ITF predicted by the model for comparison. For reference, the ITFs obtained in the previous study for a well-matched beam are also plotted in Fig. 7(b). Two effects can be observed when there is a strong curvature mismatch: the visibility of the main fringe (*λ_n_*) is reduced and secondary fringes (*S_1_^n^* and *S_2_^n^* in the figure) become apparent. These effects are due to there being less light coupling into the fundamental mode and more coupling into higher order modes [33]. The model correctly predicted the position of the secondary fringes. The model also predicted the reduction of visibility of the main fringe, although the predicted values were higher than those measured. These differences are thought to be due to alignment error.

#### 4.2.3 Defocused beam

**Fig. 8.**
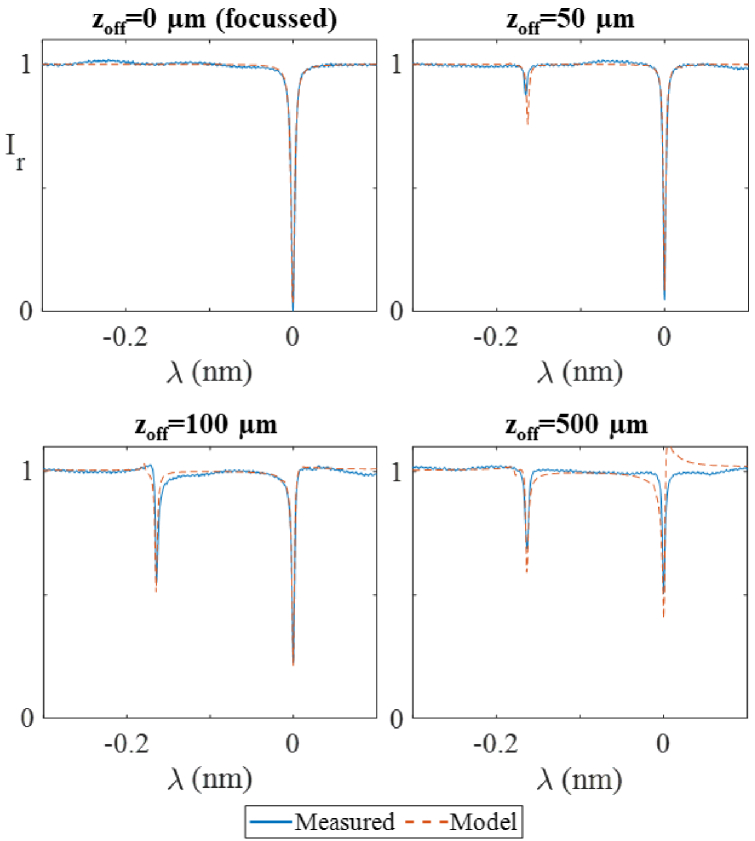
Effect of increasing distance (*z_off_*) between beam waist and planar mirror of PCMR Comparison of reflected ITFs simulated with the ABCD model (dashed lines) and experimentally measured (solid lines) when the planar mirror of the PCMR sensor is separated from the illumination focused plane by a distance *z_off_*, using 2*ω_0 _*= 38 µm.

To investigate the effect of illuminating with a de-focused beam, ITFs were measured with the beam waist located at four different axial distances *z_off_* from the planar mirror of the PCMR [Fig. 8]. Compared to the ITF obtained at the focus (*z_off _*= 0 µm), the ITFs obtained at increasing *z_off_* exhibit reduced visibility in the main fringe and contain a secondary fringe of increasing visibility. The corresponding modelled ITFs were calculated by including an extra ABCD matrix representing the additional propagation through space, as described in Ref. [25]. The model accurately predicted the position of the secondary fringes. There is also a relatively good agreement in the visibilities and shapes of the measured and predicted fringes.

## 5. Discussion

A model of light propagation in PCMRs based on the ABCD transfer matrix method has been developed. To validate the model, nearly 300 predicted ITFs of PCMRs with different reflectivities in the range 95-99.5% were compared to corresponding experimentally measured ITFs. There was a good visual agreement in the ITFs, and a low error in the FWHM, visibility and finesse. To supplement the experimental validation, in two scenarios in which the PCMR could be approximated as a planar FP etalon, the predicted ITFs were compared to those predicted by a previously validated AAF model of planar etalons. There was a good agreement between the models. Predicted and measured ITFs were also compared under conditions involving mismatched and defocused interrogation beams. Again, there was good agreement, except in the visibility of an ITF obtained with a mismatched beam, which was attributed to alignment errors.

The model could be used as a tool to aid designing and evaluating PCMRs and PCMR systems in a range of ways. For example, it could enable optimizing the sensitivity of PCMR sensors by determining which reflectivities produce the maximum ITF gradient for a specific combination of other PCMR and beam parameters. Likewise, it could enable optimizing the linear range of PCMR based frequency discriminators [34] by examining the shape of the fringes produced by different combinations of beams and PCMRs. By explicitly changing the optical elements in the model, it could also be used to help select the most appropriate lenses to deliver light to and from the PCMR to optimize the ITF, or used to estimate axial misalignment tolerances by quantifying changes in the ITF due to defocusing.

The model is relatively efficient. The computation time is proportional to the number of roundtrips. The number required is determined by the rate of convergence, and depends on the Q-factor of the PCMR. For the examples above, the number of roundtrips varied between 600 and 1300, for Q-factors between 10^5^ and 10^6^, respectively. A two-orders-of-magnitude increase in the Q-factor leads to approximately an order-of-magnitude increase in the number of required roundtrips. As well as the number of roundtrips, the computational complexity scales with the number of wavelengths of light. This depends on the wavelength range of interest and the spectral sampling rate required to accurately reproduce the shape of the fringe. Strong resonances result in low FWHMs, which require a fine sampling of the wavelength [<0.2 pm in this work, as in Fig. 5]. However, even in these demanding conditions, the maximum simulation time for any of the ITFs in this paper was <5 minutes using a standard desktop computer (64-bit operating system, Intel Core i7-9700 CPU 3.0 GHz processor, 16GB RAM). The model is therefore fast enough to be used as a practical design tool.

The model could be extended to enable studying a wider range of practically relevant scenarios in PCMRs, such as off-axis illumination. To enable this, the current ABCD model, which is based on 2-by-2 transfer matrices, could be replaced by a 3-by-3 matrix based model [35] to allow off-axis propagation of the Gaussian beam. This would enable the sensitivity of the PCMR to off-axis illumination, and the effect of off-axis misalignment tolerances, to be studied.

## 6. Conclusions

A numerical model of beam propagation through PCMR systems based on the ABCD transfer matrix method was presented. The well-established ABCD formalism provided a simple and modular representation of PCMRs, as well as the optical components that deliver light to and from them. The model is adaptable, easy to use and interpret. It is also relatively fast, typically requiring no more than a few minutes to calculate high-resolution ITFs on a standard desktop computer. To validate the model, predicted and experimentally measured ITFs of PCMRs were compared and a good agreement was observed. The validity and efficiency of the model suggest it could be used as a tool to aid the design and evaluation of PCMR systems. For example, it could find immediate application for predicting and explaining the limits of PCMR based ultrasound sensors. Straightforward extensions to the model could broaden its applicability to representing a wider range of possible conditions in these and related systems.

## Data Availability

The code is open-source and freely available in Ref. [36].
